# Differences and analogies in thyroid cancer discovered incidentally or by thyroid-related screening: a multicenter study

**DOI:** 10.1530/ETJ-24-0190

**Published:** 2025-02-04

**Authors:** Laura Croce, Rosaria Maddalena Ruggeri, Camilla Virili, Carlo Cappelli, Marsida Teliti, Pietro Costa, Spyridon Chytiris, Antonio Nicocia, Francesca Coperchini, Maria Flavia Bagaglini, Flavia Magri, Alfredo Campennì, Mario Rotondi

**Affiliations:** ^1^ Department of Internal Medicine and Therapeutics, University of Pavia, Pavia, Italy; ^2^ Istituti Clinici Scientifici Maugeri IRCCS, Unit of Endocrinology and Metabolism, Laboratory for Endocrine Disruptors, Pavia, Italy; ^3^ Department of Human Pathology and Childhood “G. Barresi” (DETEV), University of Messina, Messina, Italy; ^4^ Endocrinology Section, Department of Medico-Surgical Sciences and Biotechnologies, “Sapienza” University of Rome, Latina, Italy; ^5^ Department of Clinical and Experimental Sciences, SSD Medicina ad indirizzo Endocrino-Metabolico, University of Brescia, ASST Spedali Civili di Brescia, Brescia, Italy; ^6^ Department of Biomedical and Dental Sciences and Morpho-Functional Imaging, Nuclear Medicine Unit, University of Messina, Messina, Italy

**Keywords:** thyroid cancer, cardiovascular diseases, metabolic syndrome, incidental diagnosis, thyroid ultrasound

## Abstract

**Graphical Abstract:**

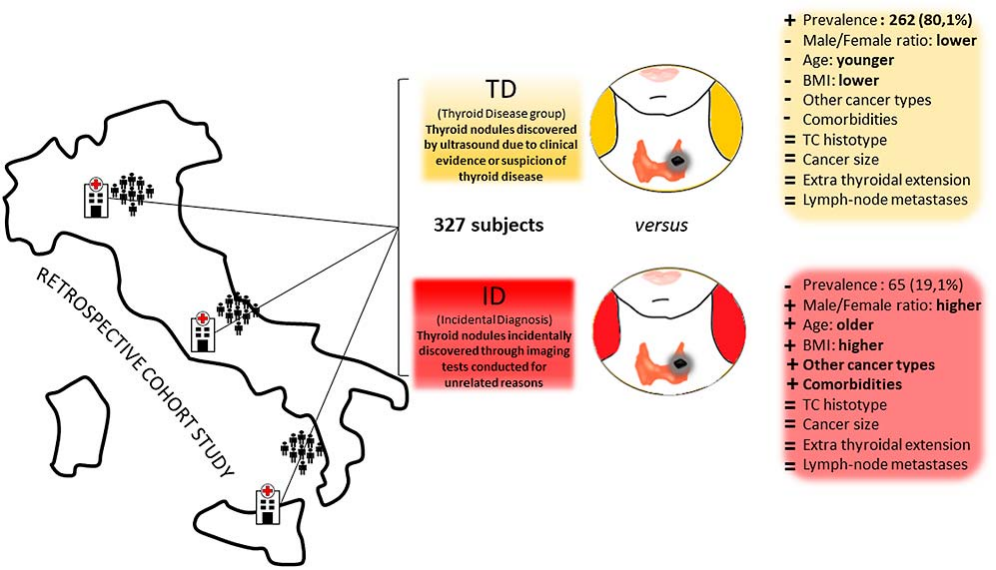

**Abstract:**

**Objective**

The prevalence of thyroid cancer (TC) has increased worldwide, and an association with metabolic and cardiovascular disorders has been reported. Moreover, an increasing percentage of patients are currently diagnosed incidentally through non-thyroid-related imaging for other clinical conditions. Our aim was to assess the prevalence of thyroid-related disease (TD) versus incidental diagnosis (ID) pre-surgery reasons leading to TC diagnosis and to compare the two groups in terms of clinical characteristics, size and severity of TC at presentation and rate of non-thyroid cancers and cardiovascular/metabolic comorbidities.

**Design**

We performed a retrospective cohort study in three high-volume hospital-based centers for thyroid diseases (Pavia, Latina and Messina) in Italy.

**Patients**

Consecutive patients with TC were included.

**Measurements**

Data on pre-surgery reasons leading to TC diagnosis, age, sex, BMI, presence of cardiometabolic comorbidities and non-thyroid cancer were collected.

**Results**

Among the 327 enrolled subjects, the diagnosis of TC was prompted by thyroid-related reasons in 262 (80.1%, TD group) and incidental in 65 (19.9%, ID group). The ID group patients were more frequently males, significantly older and with a higher BMI than the TD group ones; they had a higher rate of non-thyroidal cancers and cardiovascular/metabolic comorbidities. No significant differences could be observed in terms of TC histotype, cancer size, extrathyroidal extension, lymph node metastases, AJCC staging or American Thyroid Association (ATA) risk stratification.

**Conclusions**

Biological features of TC are similar in the TD and ID groups, but patients in the two groups display significant differences regarding their clinical features.

## Introduction

Thyroid cancer (TC) is the ninth most common cancer worldwide, with papillary TC (PTC) accounting for most cases (1). In the past decades, a sharp increase in the incidence of TC was observed (2).

Over the past four decades, the use of neck ultrasound (US) to detect thyroid masses has become widespread. Before the introduction of US, TC was diagnosed mainly because of signs and symptoms related to the presence of a neck mass and/or to thyroid dysfunction. US allows the detection of very small TC, and this has led to a sharp increase in the incidence of small TC (3). Several authors also suggest that the observed increase, among other causes, might result from the epidemics of obesity and metabolic diseases worldwide (4, 5, 6, 7). Moreover, the potential association between insulin resistance/diabetes (6, 8, 9), hypertension (10), dyslipidemia (11) and TC was suggested.

In this context, a growing number of patients receive a diagnosis of thyroid nodules (TNs) not because of the clinical suspicion of a thyroid disease, but through radiological investigations performed for non-thyroid-related reasons (12, 13). Several recent studies estimate that up to 30% of patients receiving a diagnosis of TNs do so incidentally, especially among older and male subjects (14, 15, 16), with similar data reported also in TC series (17, 18, 19, 20, 21). On the other hand, the estimated prevalence of incidentally discovered TC ranges widely between 9 and 88% (18, 19, 20, 21, 22, 23, 24, 25, 26). This high variability probably depends on screening procedures (palpation versus US and population screening) employed in different populations and on differences in definition of incidental finding. In addition, data regarding the clinical severity of TC cases are discrepant in the literature. Indeed, while some studies suggest that incidentally detected cases could be smaller and less severe (20, 24), other studies failed to show any difference (17, 18, 19, 27).

The aim of this multicenter retrospective study was to investigate pre-surgery reasons leading to the diagnosis of histologically proven TC in three endocrinological centers in the north, center and south of Italy. More in detail, the main aims were to i) assess the prevalence of patients with histologically proven TC with pre-surgery diagnosis for thyroid-related disease (TD) vs pre-surgery incidental diagnosis (ID) at imaging unrelated to thyroid disease; ii) compare the demographic, anthropometrical and clinical characteristics of patients in the TD and ID groups; and iii) compare the size and severity of TC at presentation in the ID and TD group.

## Materials and methods

The outpatients’ databases of the Endocrinology Units of Pavia (ICS Maugeri, Pavia, Italy), of Messina (Messina University Hospital, Messina, Italy) and of Latina (Sapienza University, Latina, Italy) were retrospectively searched for consecutive patients who received a diagnosis of TC between December 2021 and October 2022. To note, according to the second survey (2015–2019) conducted by the Italian National Observatory for Monitoring Iodine Prophylaxis (OSNAMI), the iodine nutritional status of the Italian population appears to be adequate and the three geographical areas do not significantly differ concerning iodine nutrition (28).

The inclusion criteria were i) histologically proven TC; ii) availability of information regarding the reason leading to TC diagnosis; iii) availability of a thyroid US before thyroid surgery at the same institution; iv) availability of fine-needle aspiration cytology (FNAC) results before surgery at the same institution; and v) age >18 years. Exclusion criteria were i) a post-surgical ID of TC at histology (i.e., in patients who had not performed FNAC or in which cancer was detected on nodules other than the one on which FNAC had been performed) and ii) lack of information regarding the reason leading to the diagnosis of TC. For the purpose of the present study, patients were then stratified according to the pre-surgery reason leading to the diagnosis of TC in two broad categories: i) patients who were referred for a thyroid US due to clinical evidence or suspicion of thyroid disease (Thyroid Disease group, TD) and ii) patients whose TNs were incidentally discovered through imaging tests conducted for unrelated reasons ID.

In detail, the TD group was identified by evidence for one or more of the following conditions: i) ongoing follow-up for thyroid nodular disease, thyroid dysfunction or thyroid autoimmunity; ii) clinically detected neck masses (either thyroid masses or nodular enlargement) or complaints of constrictive symptoms; and iii) screening procedures for family history of thyroid disease or any thyroid-related symptom.

The incidental group was identified by the discovery of TNs subsequently proven to be TC by i) a Doppler US of carotid arteries; ii) a computerized tomography (CT) or a magnetic resonance imaging (MRI), which included the neck area; iii) a PET scan; and iv) a neck US performed for non-thyroid conditions, including ear, nose and throat pathologies, neck lymph nodes enlargement and primary hyperparathyroidism.

In any case, the diagnosis of TN, subsequently classified as malignant (i.e., TC), was confirmed by a thyroid US performed by skilled endocrinologists. In each center, thyroid US scans were performed by the same experienced operator using a real-time US device equipped with a linear transducer operating at 7.5 MHz. TNs were classified according to the European Thyroid Imaging and Reporting Data System (EU-TIRADS) categories, with FNAC recommended as appropriate (29). FNAC was performed under US guidance by skilled endocrinologists and samples were classified according to the Italian consensus for thyroid cytopathology (29). The Italian classification is essentially comparable with other internationally recognized systems, particularly the Bethesda system for reporting thyroid cytology (30).

Thyroid surgery was recommended for all nodules initially classified as suspicious for or consistent with malignancy based on laboratory, clinical, US and FNAC evaluations. However, the indication for thyroid surgery differed between patients with TIR 3A and TIR 3B nodules, (corresponding to Bethesda categories III and IV, respectively). In each center, for TIR 3A/Bethesda III nodules, US surveillance is recommended. Consequently, this study included patients with TIR 3B, TIR 4 and TIR 5 cytology results (Bethesda IV, V and VI, respectively). According to the inclusion criteria, only patients with histologically proven TC entered the study. Noninvasive follicular thyroid neoplasm with papillary-like nuclear features (NIFTP) and follicular tumor with uncertain malignant potential (FT-UMP) were considered benign lesions and were not taken into account for the subsequent analysis.

At the time of diagnosis, the following data were recorded: patient’s age (years), sex (male vs female), body mass index (kg/m^2^) and a full medical history specifically focusing on the concomitant presence of non-thyroidal malignancies, type 2 diabetes mellitus, arterial hypertension, dyslipidemia, ischemic cardiopathy, cerebral vasculopathy and peripheral artery disease. Ultrasonographic data, including the presence of a single nodule or a multinodular goiter and the longest diameter of the larger malignant nodule at US, were also collected.

TCs found at histology were classified according to the 4th edition of the WHO classification (31) and staged according to the 8th edition of the American Joint Committee on Cancer (AJCC) system (32). According to the post-surgery histology, risk of persistent or recurrent PTC or follicular thyroid carcinoma (FTC) was estimated in accordance with the 2009 ATA guidelines and relevant modifications in the 2015 updated release (33, 34). In detail, the information considered for ATA risk stratification were: presence or absence of extrathyroidal extension (ETE), vascular invasion, aggressive histological subtypes (i.e., tall-cells, hob-nail, sclerosing variants) and presence, number and size of neck lymph node metastases.

All procedures were in accordance with the ethical standards of the institutional research committee and with the 1964 Helsinki declaration and its later amendments. The study was approved by the Ethical Committee of ICS Maugeri, Pavia (Protocol N. 2742 CE). All subjects enrolled have provided written consent after receiving a full explanation regarding the future use of their clinical data for research purposes.

### Statistical analysis

Statistical analysis was performed using the SPSS (SPSS, Inc.; https://www.ibm.com/products/spss-statistics?lot=5&mhsrc=ibmsearch_a&mhq=spss). Between-group comparisons were performed using Student’s *t*-test for unpaired data and the Mann–Whitney U test according to a normal or a non-parametric distribution. Within-group comparisons were performed using Student’s *t*-test for paired data and the Wilcoxon test according to a normal or a non-parametric distribution. Frequencies among groups were performed using the χ2-test with Fisher’s correction when appropriate. The required sample size was calculated considering i) a type I error of 0.01 and a type II error of 0.20; ii) an expected prevalence of ID of TC of around 20% among our series of patients (based on preliminary data); and iii) an expected prevalence of cardiovascular/metabolic comorbidities of around 60% in the ID group compared with 30% of the non-ID group. The required sample size was of at least 258 patients.

## Results

**Figure 1:**
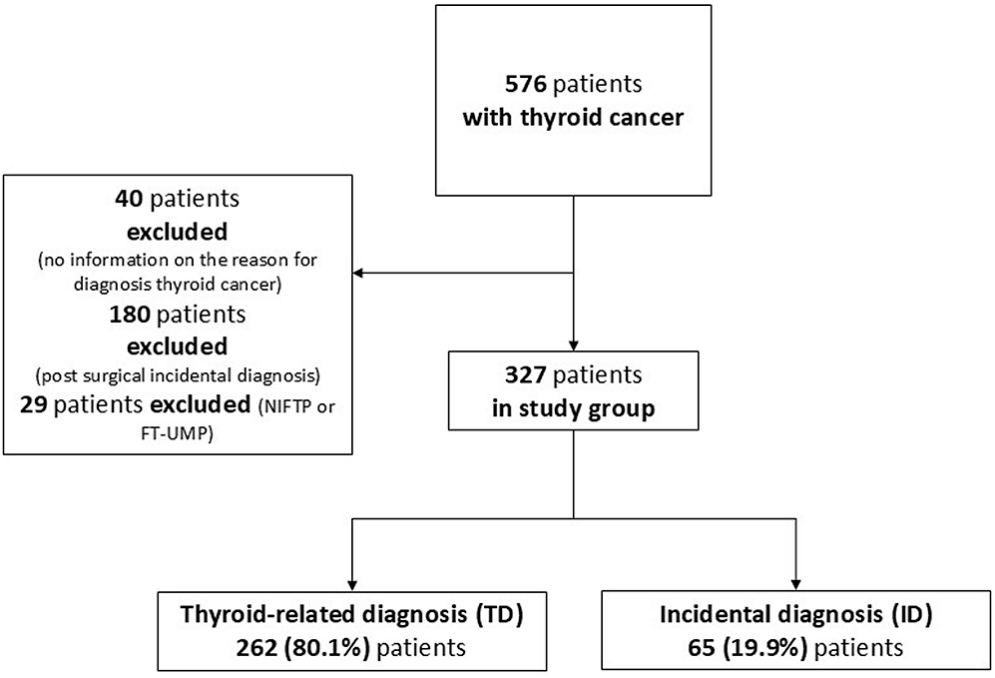
Flow chart of the study.

As shown in Fig. 1, according to the exclusion and inclusion criteria, a total of 327 patients with a histological diagnosis of TC were enrolled. The TD group included 262 patients (80.1%), and the ID group included 65 patients (19.9%). More in detail, among the TD group, 77 patients (29.4%) were followed up for a thyroid dysfunction, 73 (27.9%) were followed-up for a nodular goiter, 40 (15.3%) had a clinically detected neck mass, 13 (4.9%) complained of dysphagia/dysphonia, 32 (12.2%) were screened for a family history of thyroid diseases, and 27 (10.3%) were screened for other reasons.

In the TD group, 53 out of 262 patients had a clinically detected neck mass and/or constrictive symptoms. The former showed larger cancer both at US (median size 20 (12.5–36) vs 12 (9–19) mm, *P* < 0.001) and at histology (13 (8–21.2) vs 9 (7–14) mm, *P* = 0.001)) and had a higher rate of high risk according to the ATA risk classification (7.7 vs 1.5%, *P* = 0.021) as compared to the latter. No significant differences were observed as to age, sex, BMI, rate of comorbidities, rate of multinodular goiter, histotype, ETE, lymph node metastasis and AJCC staging.

Among the ID group, 28 patients (43.1%) had a Doppler US of carotid arteries, 24 (36.9%) had a neck US for non-thyroid-related reasons, 6 (9.2%) had a CT scan, 6 (9.2%) had a PET, and 1 (1.6%) had an MRI.

None of the patients who underwent neck US due to cervical lymph node enlargement were ultimately diagnosed with metastasis in the clinically detected lymph node.

As summarized in Table 1, patients in the ID group had a significantly higher male-to-female ratio, were significantly older, had a higher BMI and had a significantly higher rate of cardiovascular comorbidities, when compared to the TD group. Moreover, a higher rate of non-thyroid malignancies was observed in the ID group. On the other hand, while the rate of multinodular goiter was higher in the TD group, the maximum diameter of TN at US and FNAC results were similar in the two groups.

**Table 1:** Comparison between patients with an incidentally discovered thyroid cancer (ID) and patients diagnosed for thyroid-related reasons (TD). Data are presented as mean ± SD or as *n* (%). Bold text represents *P <* 0.05.

	TD	ID	*P* value
*N*	262	65	
Age (years)	47.2 ± 14.1	53.3 ± 15.8	**0.003**
Sex males/females (% of males)	60/202 (22.9%)	32/33 (49.2%)	**<0.001**
BMI (kg/m^2^)	25.8 ± 4.8	27.8 ± 6.2	**0.006**
Patients with cardiovascular/metabolic comorbidities	87 (33.2%)	39 (60.0%)	**<0.001**
Other malignancies	28 (10.7%)	14 (21.5%)	**0.020**
Maximum diameter of the largest cancerous nodule at US (mm, median)	13 (9–20)	13 (11–20)	0.582
Multinodular goiter	157 (59.9%)	30 (46.2%)	**0.045**
FNA			**0.026**
TIR 3B	96 (37.1%)	16 (24.6%)	
TIR 4	74 (28.6%)	15 (23.1%)	
TIR 5	89 (34.3%)	34 (52.3%)	
Total thyroidectomy	244 (93.1%)	59 (90.8%)	0.514
Histotype			0.656
PTC	240 (91.6%)	59 (90.8%)	
FTC	12 (4.6%)	2 (3.1%)	
MTC	9 (3.4%)	3 (4.6%)	
PDC	1 (0.4%)	1 (1.5%)	
Maximum diameter at histology (mm, median)	9.5 (7.0–15.0)	10.0 (7.0–15.5)	0.171
Multifocal	91 (34.7%)	18 (27.7%)	0.281
ETE	89 (34.0%)	30 (46.2%)	0.068
Lymph node metastasis	53 (20.2%)	15 (23.1%)	0.613
T stages			0.650
T1a	129 (49.2%)	25 (38.5%)	
T1b	88 (33.6%)	26 (40.0%)	
T2	33 (12.6%)	10 (15.4%)	
T3a	9 (3.4%)	3 (4.6%)	
T3b	3 (1.2%)	1 (1.5%)	
T4a	0 (0.0%)	0 (0.0%)	
T4b	0 (0.0%)	0 (0.0%)	
AJCC stage			0.804
I	232 (88.5%)	56 (86.2%)	
II	28 (10.7%)	9 (13.8%)	
III	0 (0.0%)	0 (0.0%)	
IV A	1 (0.4%)	0 (0.0%)	
IV B	1 (0.4%)	0 (0.0%)	
ATA risk*			0.826
Low risk	160 (70.2%)	38 (69.1%)	
Intermediate risk	63 (27.6%)	15 (27.3%)	
High risk	5 (2.2%)	2 (3.6%)	

AJCC, American Joint Committee on Cancer; ATA, American Thyoid Association; BMI, body mass index; FNA, fine-needle aspiration; IQR, interquartile range, PTC, papillary thyroid carcinoma; FTC, follicular thyroid carcinoma, MTC, medullary thyroid carcinoma; PDC, poorly differentiated carcinoma; US, ultrasound; TD, thyroid-related disease; ID, incidental diagnosis; ETE, extrathyroidal extension.

Regarding post-surgical data, no significant differences could be observed in terms of surgical approach (thyroidectomy vs lobectomy), TC histotype, cancer size at histology, ETE, lymph node metastases, AJCC staging or ATA risk stratification.

## Discussion

The results of the present study show that, among a cohort of patients with histologically proven malignant TC coming from three different areas of Italy, ID through thyroid-unrelated imaging accounts for approximately 20% of all cases. The most frequent cause of incidental finding in our series was Doppler US of carotid arteries, accounting for almost 50% of cases. Patients with an ID were older and more frequently males and had a higher BMI and a higher rate of non-thyroidal malignancies, cardiovascular and metabolic comorbidities. On the other hand, no significant differences could be observed in terms of TC size at histology, histotype, extrathyroidal extension, lymph node metastases, AJCC staging and ATA risk stratification.

The impact of ID of TC was assessed by several previous studies, including a recent study by Cosme *et al.* (17, 18, 19, 20, 35). However, the present study highlights some novel aspects.

The definition of ‘ID’ of TC varies across studies. Some studies include post-surgical, histologically detected cases (18, 24), and some other consider cases detected during screening for thyroid diseases as incidental (18, 20, 26). In particular, in the recent study by Cosme *et al.*, a broader definition of ID is employed, including cases discovered through screening procedures, detected in patients with thyroid dysfunction or incidentally identified during postoperative histological examinations (35). According to the purpose of the present study, the term ‘incidental’ refers to any thyroid carcinoma discovered during imaging procedures for reasons unrelated to the thyroid, focusing on patients who otherwise would not have been referred to an endocrinologist. The present study shows that this particular category of patients represents a relevant part of the TC population. The present study includes all histotypes of TC, not only PTC. This comprehensive inclusion provides a broader perspective on the ID of TC, allowing us to show that ID impacts equally the different histotypes of TC.

The results obtained demonstrate that, at least in Italy, Doppler US of carotid arteries is currently the primary imaging procedure leading to incidental TC diagnosis. In previous studies, chest CT scans were the most commonly employed techniques (18, 19). This finding is particularly significant, as it highlights that incidental TC diagnoses now predominantly involve subjects with a dysmetabolic phenotype, as evidenced by the high rate of cardiovascular and metabolic comorbidities observed in this group. This shift has important implications for understanding potential epidemiological associations between thyroid carcinoma and cardiovascular or metabolic conditions. Indeed, any association study between TC and cardiovascular/metabolic condition should consider the way of detection as a possible interfering factor. Moreover, ID patients have a higher rate of non-thyroid malignancies. These findings imply that any consideration on the overall survival of patients with TC should take carefully into account the way TC was diagnosed (ID versus TD), which may be related with a higher or lower risk of morbidity and mortality due to concurrent causes. This statement appears to be in keeping with previous studies showing that TC mortality is more dependent on other concurrent conditions than on TC per se (36).

We observed similar severity of TC between incidental and non-incidental cases. Previous studies reported contrasting results, with some studies showing a lower severity of incidental cases (18, 26) and others similar severity (19, 24). Discrepancies may be due to different definitions of ID, as previously stated. Moreover, most studies were performed before the routine use of the latest ATA guidelines and AJCC staging, which have led to relevant changes in the scoring of TC severity. Interestingly, the study by Cosme *et al.*, which was based on current guidelines, showed a similar severity between incidental and non-incidental cases (35). In line with what was noted in the two commentaries to the study by Cosme *et al.*, this suggests that incidental cases are not necessarily less severe than non-incidental ones. Incidental cases should not be regarded simply as ‘overdiagnosis’ but may include severe TC cases that require medical attention (37, 38).

Major strengths of the present study are i) the multicentric design, involving three Italian referral centers for thyroid diseases management, providing comprehensive and reliable data for the clinical picture of TC population in our country, and ii) the real-life scenario of the study, which assesses TC presentation in regular clinical practice.

The major limitations of this study are mainly related to its retrospective design and the possibility that the advent of COVID-19 may have partially impacted our data, even though the study period does not include 2020, the year in which the pandemic escalated. Indeed, the effect of COVID-19 could have been dual: a delay in TD diagnosis for non-hospitalized patients and an increase in ID diagnosis for hospitalized patients with SARS-CoV-2 infection through radiological investigations performed for non-thyroid-related reasons (e.g., chest CT) (39). However, it should be highlighted that the results of the present study are in overall agreement with the ones reported by Cosme *et al.* performed before the COVID-19 period (35).

In conclusion, this study examines the clinical presentation, tumor characteristics and comorbidities of TC patients within a real-life scenario, focusing on how these factors vary based on the diagnostic pathway. The results reported here highlight the influence of non-traditional endocrinological diagnostic pathways on the clinical and demographic profiles of TC patients. Doppler US of carotid arteries has emerged as the leading diagnostic tool for incidental TC, at least in Italy, replacing chest CT as reported in previous studies. Our results demonstrate an overall similarity as to the biological features of TC independently of how the diagnosis is reached (TD versus ID). However, patients with TC display significant differences as to their clinical history. We conclude that TC is the same, but patients are strikingly different in relation to a TD or ID diagnosis.

## Declaration of interest

The authors  declare that there is no conflict of interest that could be perceived as prejudicing the impartiality of the research reported.

## Funding

This work was partially supported by the ‘Ricerca Corrente’ funding scheme of the Ministry of Health, Italy.

## Author contribution statement

LC, RMR, CV, CC and AC conceived the study. LC, RMR, CV, CC, AN and AC designed the methodology. Data curation was done by LC, MT, SC, FC and FM. LC, PC, FC and MT wrote the manuscript. LC, PC and MT prepared the original draft. LC, RMR, CV, CC, MFB, FM, AC and MR were involved in visualization and investigation. LC, RMR, AC and MR supervised the study and reviewed and edited the manuscript.

## References

[1] Pizzato M, Li M, Vignat J, et al. The epidemiological landscape of thyroid cancer worldwide: GLOBOCAN estimates for incidence and mortality rates in 2020. Lancet Diabetes Endocrinol 2022 10 264–272. (10.1016/s2213-8587(22)00035-3) 35271818

[2] Dal Maso L, Panato C, Franceschi S, et al. The impact of overdiagnosis on thyroid cancer epidemic in Italy, 1998–2012. Eur J Cancer 2018 94 6–15. (10.1016/j.ejca.2018.01.083) 29502036

[3] Haymart MR, Banerjee M, Reyes-Gastelum D, et al. Thyroid ultrasound and the increase in diagnosis of low-risk thyroid cancer. J Clin Endocrinol Metab 2019 104 785–792. (10.1210/jc.2018-01933) 30329071 PMC6456891

[4] Kim J, Gosnell JE & Roman SA. Geographic influences in the global rise of thyroid cancer. Nat Rev Endocrinol 2020 16 17–29. (10.1038/s41574-019-0263-x) 31616074

[5] Kwon H, Chang Y, Cho A, et al. Metabolic obesity phenotypes and thyroid cancer risk: a cohort study. Thyroid 2019 29 349–358. (10.1089/thy.2018.0327) 30648486

[6] Huang L, Feng X, Yang W, et al. Appraising the effect of potential risk factors on thyroid cancer: a mendelian randomization study. J Clin Endocrinol Metab 2022 107 e2783–e2791. (10.1210/clinem/dgac196) 35366326

[7] Fussey JM, Beaumont RN, Wood AR, et al. Does obesity cause thyroid cancer? A mendelian randomization study. J Clin Endocrinol Metab 2020 105 e2398–e2407. (10.1210/clinem/dgaa250) 32392279 PMC7274488

[8] Duran AO, Anil C, Gursoy A, et al. The relationship between glucose metabolism disorders and malignant thyroid disease. Int J Clin Oncol 2013 18 585–589. (10.1007/s10147-012-0435-3) 22752254

[9] Greco A, Coperchini F, Croce L, et al. Drug repositioning in thyroid cancer treatment: the intriguing case of anti-diabetic drugs. Front Pharmacol 2023 14 1303844. (10.3389/fphar.2023.1303844) 38146457 PMC10749369

[10] Li LR, Song JL, Liu HQ, et al. Hypertension was associated with higher tumor stages in papillary thyroid cancer: a large sample single-center study. Metab Syndr Relat Disord 2022 20 466–472. (10.1089/met.2022.0033) 36083278

[11] Pasqual E, O'Brien K, Rinaldi S, et al. Obesity, obesity-related metabolic conditions, and risk of thyroid cancer in women: results from a prospective cohort study (Sister Study). Lancet Regional Health Americas 2023 23 100537. (10.1016/j.lana.2023.100537) 37346380 PMC10279535

[12] Drake T, Gravely A, Westanmo A, et al. Prevalence of thyroid incidentalomas from 1995 to 2016: a single-center, retrospective cohort study. J Endocr Soc 2019 4 bvz027. (10.1210/jendso/bvz027) 31993553 PMC6977946

[13] Park S, Oh CM, Cho H, et al. Association between screening and the thyroid cancer “epidemic” in South Korea: evidence from a nationwide study. BMJ 2016 355 i5745. (10.1136/bmj.i5745) 27903497 PMC5130923

[14] Croce L, Ruggeri RM, Cappelli C, et al. Cardiovascular and metabolic comorbidities in patients with thyroid nodules: the impact of incidental diagnosis. J Endocrinol Invest 2024 47 827–832. (10.1007/s40618-023-02191-4) 37702926 PMC10965599

[15] Kennedy E, Zhang Y, Qadadha Y, et al. Rates of detecting thyroid nodules recommended for biopsy with ultrasound: are all indications equal? Thyroid 2023 33 1434–1440. (10.1089/thy.2023.0234) 37981778 PMC10714116

[16] Soto Jacome C, Segura Torres D, Fan JW, et al. Drivers of thyroid ultrasound use: a retrospective observational study. Endocr Pract 2023 29 948–954. (10.1016/j.eprac.2023.09.006) 37722595 PMC10843084

[17] Kahn C, Simonella L, Sywak M, et al. Pathways to the diagnosis of thyroid cancer in New South Wales: a population-based cross-sectional study. Cancer Causes Control 2012 23 35–44. (10.1007/s10552-011-9852-2) 22002623

[18] Malone MK, Zagzag J, Ogilvie JB, et al. Thyroid cancers detected by imaging are not necessarily small or early stage. Thyroid 2014 24 314–318. (10.1089/thy.2012.0651) 23819462

[19] Yoo F, Chaikhoutdinov I, Mitzner R, et al. Characteristics of incidentally discovered thyroid cancer. JAMA Otolaryngol Head Neck Surg 2013 139 1181–1186. (10.1001/jamaoto.2013.5050) 24113885

[20] Wu L, Vaccarella S, Feng CY, et al. Mortality among papillary thyroid cancer patients by detection route: a hospital-based retrospective cohort study. Eur Thyroid J 2023 12 e230127. (10.1530/etj-23-0127) 37855414 PMC10692677

[21] Norwood TA, Buajitti E, Lipscombe LL, et al. Incidental detection, imaging modalities and temporal trends of differentiated thyroid cancer in Ontario: a population-based retrospective cohort study. CMAJ Open 2020 8 E695–E705. (10.9778/cmajo.20200095) PMC7608946 33139390

[22] Brito JP, Al Nofal A, Montori VM, et al. The impact of subclinical disease and mechanism of detection on the rise in thyroid cancer incidence: a population-based study in olmsted county, Minnesota during 1935 through 2012. Thyroid 2015 25 999–1007. (10.1089/thy.2014.0594) 26103159 PMC4560845

[23] Zagzag J, Malone MK, Lopresti MA, et al. Method of detection of well-differentiated thyroid cancers in obese and non-obese patients. PLoS One 2016 11 e0152768. (10.1371/journal.pone.0152768) 27043928 PMC4820112

[24] Marina M, Ceda GP, Aldigeri R, et al. Causes of referral to the first endocrine visit of patients with thyroid carcinoma in a mildly iodine-deficient area. Endocrine 2017 57 247–255. (10.1007/s12020-016-1140-1) 27738889

[25] Toyoda Y, Tabuchi T, Nakata K, et al. Increase in incidental detection of thyroid cancer in Osaka, Japan. Cancer Sci 2018 109 2310–2314. (10.1111/cas.13645) 29788541 PMC6029814

[26] Kim SH, Roh JL, Gong G, et al. Differences in the recurrence and survival of patients with symptomatic and asymptomatic papillary thyroid carcinoma: an observational study of 11,265 person-years of follow-up. Thyroid 2016 26 1472–1479. (10.1089/thy.2016.0238) 27457917

[27] Chooi JE, Ravindiran A & Balasubramanian SP. The influence of incidental detection of thyroid nodule on thyroid cancer risk and prognosis-A systematic review. Clin Endocrinol 2022 96 246–254. (10.1111/cen.14575) 34378225

[28] Olivieri A, Andò S, Bagnasco M, et al. The iodine nutritional status in the Italian population: data from the Italian national observatory for monitoring iodine prophylaxis (OSNAMI) (period 2015–2019). Am J Clin Nutr 2019 110 1265–1266. (10.1093/ajcn/nqz206) 31667512

[29] Nardi F, Basolo F, Crescenzi A, et al. Italian consensus for the classification and reporting of thyroid cytology. J Endocrinol Invest 2014 37 593–599. (10.1007/s40618-014-0062-0) 24789536

[30] Cibas E S & Ali S Z. The 2017 Bethesda System for Reporting Thyroid Cytopathology. Thyroid 2017 27 1341–1346. (10.1089/thy.2017.0500) 29091573

[31] Lloyd RV, Osamura RY, Klöppel G, et al. WHO Classification of Tumors of Endocrine Organs, vol 10, 4th edn. Lyon, France: International Agency for research on Cancer. (https://www.iarc.who.int/news-events/who-classification-of-tumours-of-endocrine-organs/)

[32] Tuttle RM, Haugen B & Perrier ND. Updated American Joint committee on cancer/tumor-node-metastasis staging system for differentiated and anaplastic thyroid cancer (eighth edition): what changed and why? Thyroid 2017 27 751–756. (10.1089/thy.2017.0102) 28463585 PMC5467103

[33] Haugen BR, Alexander EK, Bible KC, et al. 2015 American thyroid association management guidelines for adult patients with thyroid nodules and differentiated thyroid cancer: the American thyroid association guidelines task force on thyroid nodules and differentiated thyroid cancer. Thyroid 2016 26 1–133. (10.1089/thy.2015.0020) 26462967 PMC4739132

[34] Haugen BR, Sawka AM, Alexander EK, et al. American thyroid association guidelines on the management of thyroid nodules and differentiated thyroid cancer task force review and recommendation on the proposed renaming of encapsulated follicular variant papillary thyroid carcinoma without invasion to noninvasive follicular thyroid neoplasm with papillary-like nuclear features. Thyroid 2017 27 481–483. (10.1089/thy.2016.0628) 28114862

[35] Cosme I, Figueiredo A, Pinheiro S, et al. Incidentally vs non-incidentally diagnosed papillary thyroid carcinoma: are there differences? Eur Thyroid J 2024 13 e240106. (10.1530/ETJ-24-0106) 38968008 PMC11301558

[36] Papaleontiou M, Norton EC, Reyes-Gastelum D, et al. Competing causes of death in older adults with thyroid cancer. Thyroid 2021 31 1359–1365. (10.1089/thy.2020.0929) 33764188 PMC8591088

[37] Cosme I, Figueiredo A, Pinheiro S, et al. Incidental thyroid cancer and overdiagnosis: response to drs tsybrovskyy, sobrinho-simões, and tallini. Eur Thyroid J 2024 13 e240296. (10.1530/etj-24-0296) 39400058 PMC11558922

[38] Tsybrovskyy O, Sobrinho-Simões M & Tallini G. Incidental thyroid cancer' is not synonymous with 'overdiagnosis. Eur Thyroid J 2024 13 e240283. (10.1530/etj-24-0283) 39400060 PMC11558969

[39] Helvacı BC, Ozdemir D, Turan K, et al. Incidental thyroid nodules on COVID-19-related thoracic tomography scans: a giant cohort. Hormones 2024 23 227–233. (10.1007/s42000-023-00516-9) 38103164

